# Development of a Purity Certified Reference Material for Vinyl Acetate

**DOI:** 10.3390/molecules28176245

**Published:** 2023-08-25

**Authors:** Chen He, Qin Gao, Changwen Ye, Guotao Yang, Pengfei Zhang, Rongchao Yang, Qing Zhang, Kang Ma

**Affiliations:** 1 Zhengzhou Tobacco Research Institute of China National Tobacco Corporation, Zhengzhou 450001, China; hechen2012@sina.com (C.H.); mtjj20200601@163.com (Q.G.); yectsrc@163.com (C.Y.); ygt3315@163.com (G.Y.); yczhangpf@163.com (P.Z.); yangrongchao2760@126.com (R.Y.); 2 Division of Chemical Metrology and Analytical Science, National Institute of Metrology, Beijing 100013, China

**Keywords:** vinyl acetate, mass balance method, purity, certified reference materials, uncertainty

## Abstract

Vinyl acetate is a restricted substance in food products. The quantification of the organic impurities in vinyl acetate is a major problem due to its activity, instability, and volatility. In this paper, while using the mass balance method to determine the purity of vinyl acetate, an improved method was established for the determination of the content of three impurities in vinyl acetate reference material, and the GC-FID peak area normalization for vinyl acetate was calibrated. The three trace organic impurities were identified by gas chromatography tandem high-resolution mass spectrometry to be methyl acetate, ethyl acetate, and vinyl propionate. The content and relative correction factors for the three organic impurities were measured. The purity of vinyl acetate determined by the mass balance method was 99.90% with an expanded uncertainty of 0.30%, and the total content of organic impurities was 0.08% with a relative correction factor of 1.23%. The vinyl acetate reference material has been approved as a national certified reference material in China as GBW (E) 062710.

## 1. Introduction

Vinyl acetate is a colorless, flammable liquid with a sweet, ether flavor. Because of the presence of carbon–carbon double bonds, vinyl acetate is active, and chemical reactions, such as addition and polymerization reactions, are prone to occurring [1]. In industry, vinyl acetate is mainly used as a polymer or copolymer monomer in the synthesis of polyvinyl acetate homopolymer emulsions, vinyl acetate ethylene copolymer emulsions, ethylene vinyl alcohol copolymer emulsions, and other common adhesives that are widely used in the food, construction, and other industries [2,3,4,5]. Vinyl acetate is irritating to the eyes, skin, mucous membranes, and upper respiratory tract, and long-term exposure can anesthetize nerves. It has been proven that vinyl acetate is carcinogenic to animals, and it is classified as a class 2B carcinogen [6,7]. The food, construction, and other industries in China have restrictions on the allowable content of vinyl acetate in specific industrial products [8]. Therefore, analytical methods for the accurate measurement of vinyl acetate are required in order to provide quality assurance for commercial products. Currently, the reported analysis methods include the saponification [9], thermo-gravimetric [10], nuclear magnetic resonance (NMR) spectroscopy [11], infrared spectroscopy [12], and gas chromatography (GC) [13] methods. Because of its high accuracy and sensitivity, chromatography is a common method for the quantitative determination of vinyl acetate, and several chromatographic methods have been reported for the quantitative determination of vinyl acetate in food contact materials [14], plastics [15], and white latex [16]. However, chromatographic quantification usually requires a certified reference material (CRM) to prepare a standard solution. Therefore, it is necessary to establish a measurement method for a vinyl acetate CRM and to use this vinyl acetate CRM to make the currently used methods more accurate and convenient.

There have been few reports of purity determination methods for vinyl acetate. Accurate purity determination methods are the basis for the development of CRMs, and play an important role in the establishment of measurement traceability, the calibration of instruments, and verification methods [17,18], Therefore, CRMs have important uses in food, medicine, and other fields [19,20,21].

In the development of CRMs, the quantitative nuclear magnetic resonance (q-NMR) [22,23], thermal analysis [24,25], and mass balance [26,27,28,29] methods are commonly used methods for measuring the purity of organic substances. The mass balance method is generally considered to be a relatively high-precision method for purity determination, and can be directly traced back to the SI units of mass (kg) and amount of substance (mol). The mass balance method is one of the main methods for determining the purity of substances in the Organic Analysis Work Group of the International Bureau of Weights and Measures [30]. The mass balance method is also one of the methods for determining the purity of pharmaceutical reference substances that is recommended by the World Health Organization and the European Pharmacopoeia [31,32]. Mass balance, high performance liquid chromatography (HPLC), and differential scanning calorimetry (DSC) were used by Kang Ma et al. to determine the purity of a theophylline CRM, and the accuracy of the mass balance method was shown to be better than that of the other methods [33]. With the mass balance method, HPLC and GC are frequently used to measure organic components. A mass balance method combined with gas chromatography–mass spectrometry was used by Wang et al. [34] to determine the content of benzene and to investigate the accuracy of DSC and q-NMR measurements. The mass balance method was used by Chen et al. to assign purity to four unsaturated fatty acid esters; the gas chromatographic area normalization method was used to determine the content of the main components; and the content of volatile impurities, moisture, and non-volatile impurities was calculated [35]. However, because of the nonlinear response of the detector, the percentages calculated from the peak areas were not equal to the percentages of each component. Therefore, a correction factor was introduced by Wang et al. to correct the values for the purity obtained by area normalization to produce more accurate measurement results [36].

However, it is still a challenge to determine vinyl acetate’s purity using the mass balance method, which can mainly be attributed to the difficulty of identifying and quantifying the organic impurities in vinyl acetate. This difficulty occurs because vinyl acetate and the organic impurities in it have small molecular weights and are difficult to separate.

Therefore, in the present study, a new pure CRM for vinyl acetate was developed, and a mass balance method was established to determine the purity of the vinyl acetate. The gas chromatographic area normalization results were corrected and recalculated by characterizing and calculating the relative correction factors for three organic impurities in vinyl acetate. In addition, a homogeneity test and stability study were carried out on the prepared vinyl acetate CRM, and the uncertainty of the CRM was evaluated.

## 2. Results and Discussion

### 2.1. Characterization of the CRM Candidate

#### 2.1.1. Mass Spectrometry (MS) Analysis

**Figure 1:**
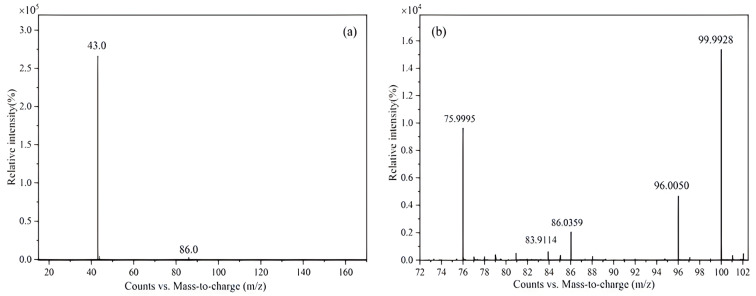
Mass spectrometry results of CRM candidate: (**a**) low-resolution mass spectrum; (**b**) high-resolution mass spectrum (*m*/*z* 75.9995, *m*/*z* 96.0050, and *m*/*z* 99.9928 are the molecular ion peaks of the reference ions).

The mass spectrometry analysis of the CRM candidate was performed in positive ion mode, and the results are shown in Figure 1. Figure 1a shows the low-resolution spectrum. According to the molecular structure of vinyl acetate, the molecular ion [CH_3_COOCH=CH_2_]^+^ undergoes homolytic and heterolytic decomposition under the bombardment of electrons, forming the fragment ion CH_3_C=O^+^. Therefore, the strong peak at *m*/*z* = 43 corresponded to the fragment ion CH_3_C=O^+^.

High-resolution mass spectrometry was used to accurately measure the molecular weights of the compounds, and the results are shown in Figure 1b. *m*/*z* = 86.0395 corresponded to the molecular ion peak for vinyl acetate. The measured molecular weight of vinyl acetate was detected to be 86.0359, with a theoretical value of 86.0362 and a relative analytical error of 3.85 × 10^−6^, which can be considered to be consistent with C_4_H_6_O_2_.

#### 2.1.2. Fourier Transform Infrared Spectroscopy (FT-IR) Analysis

**Figure 2:**
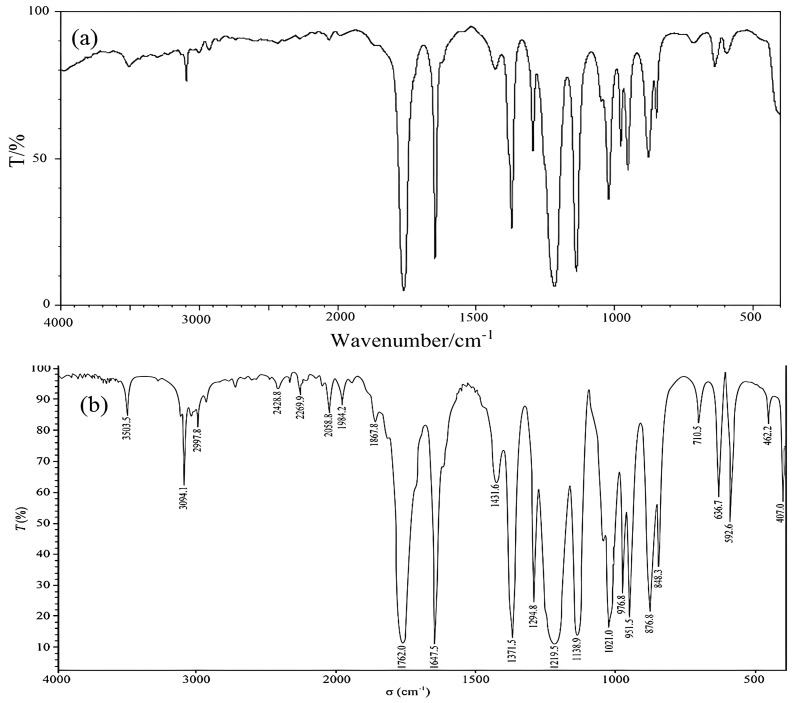
Infrared spectra of vinyl acetate: (**a**) infrared standard spectrum of vinyl acetate; (**b**) infrared spectra of CRM candidate.

Figure 2 shows the infrared spectrum of a CRM candidate. It can be seen from Figure 2 that the absorption peaks at 3094.1 and 1431.6 cm^−1^ are the C-H stretching and bending vibrations, respectively, in the carbon–carbon double bond, and the absorption peak at 1647.5 cm^−1^ is the C-C stretching vibration of the carbon–carbon double bond. This result indicates that there may be an alkene structure in the molecule. The absorption peak at 1762.0 cm^−1^ is the C=O stretching vibration of a carboxylate, and the absorption peaks at 1219.5 and 1021.0 cm^−1^ are the C-O stretching vibrations in a carboxylate, indicating that there may be a carboxylate structure in the molecule. The absorption peak at 1371.5 cm^−1^ is the C-H bending vibration of a methyl group, indicating that there may be a methyl structure in the molecule. Comparing Figure 2b with the standard infrared spectra of vinyl acetate indicates that the spectra were basically consistent.

#### 2.1.3. NMR Analysis

**Figure 3:**
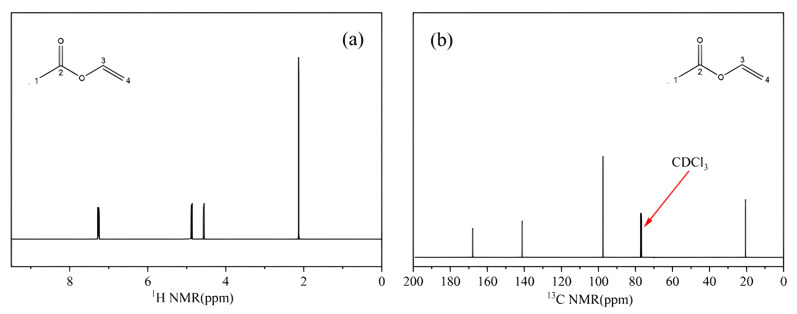
NMR spectra of CRM candidate: (**a**) ^1^H NMR spectra, (**b**) 13C NMR spectra.

Figure 3a shows the ^1^H NMR spectrum of a CRM candidate. The chemical shifts (δ, ppm) of the four proton signals were as follows: δ 7.26 (1H, dd, *J =* 6.0, 14.0 Hz) was the proton signal of C3; δ 4.88 (1H, dd, *J =* 1.5, 14.0 Hz) and δ 4.56 (1H, dd, *J =* 2.0, 6.2 Hz) were the proton signals of C4 in the carbon–carbon double bond; and δ 2.13 (3H, s) was the proton signal of the methyl group.

Figure 3b shows the chemical shift (δ, ppm) of the carbon signal: δ 77.02 was the carbon signal of the solvent CDCl_3_, δ 167.89 was the carbon signal for the carbonyl group (C2); δ 141.15 and 97.50 were the double-bonded carbon signals, δ 141.15 corresponded to the tertiary C3, δ 97.50 corresponded to the secondary C4, and δ 20.54 was the carbon signal of the methyl group (C1). According to the above analysis, the results were basically consistent with vinyl acetate.

### 2.2. Purity Determination by the Mass Balance Method

#### 2.2.1. Qualitative Analysis of Organic Impurities

**Figure 4:**
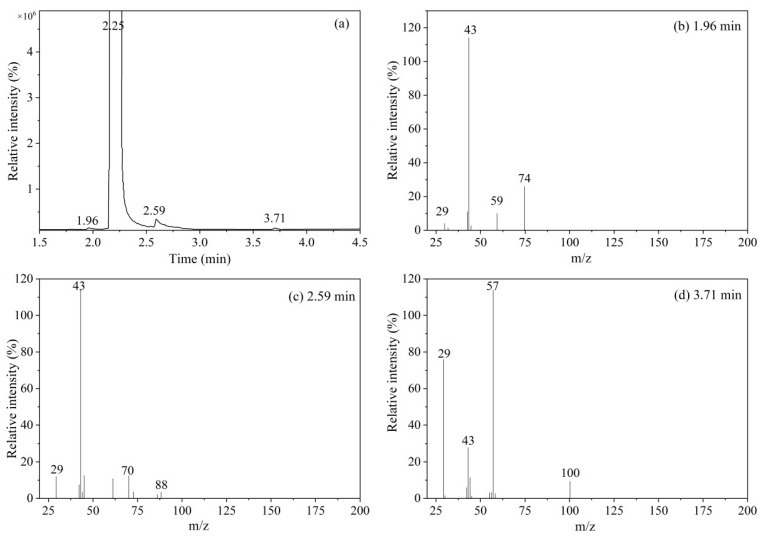
MS-TOF analysis results: (**a**) total ion chromatogram of CRM candidate; (**b**) mass spectrum of impurity #1; (**c**) mass spectrum of impurity #2; and (**d**) mass spectrum of impurity #3.

The organic impurities in the vinyl acetate CRM candidate were analyzed by MS-TOF, and the results are shown in Figure 4.

Figure 4a shows the total ion chromatogram (TIC) of a vinyl acetate candidate. It can be seen from the figure that there are four components in the candidate: the main peak at 2.25 min is vinyl acetate and the peaks at 1.96, 2.59, and 3.71 min are three organic impurities, numbered 1#, 2#, and 3#, respectively. The three impurities were analyzed by high-resolution mass spectrometry, and the results are shown in Figure 4b–d. The following conclusions can be drawn in accordance with Table 1: (1) In Figure 4b, *m*/*z* 74 is the molecular ion peak of CH_3_COOCH_3_, *m*/*z* 59 is the free radical CH_3_COO^+^ formed after [CH_3_COOCH_3_]^+^ is cleaved to remove CH_3_^•^, *m*/*z* 43 is the ion peak of CH_3_C≡O^+^ formed after cleavage, and *m*/*z* 29 is the ion peak of CH_3_CH_2_^•^ cleavage; therefore, impurity #1 may be methyl acetate. (2) In Figure 4c, *m*/*z* 88 is the molecular ion peak of CH_3_COOCH_2_CH_3_, and *m*/*z* 70, *m*/*z* 43, and *m*/*z* 61 are the fragment ions HC≡COCH_2_CH_3_^+^, CH_3_COOH_2_^+^, and CH_3_C≡O^+^, respectively, formed after cleavage and rearrangement of the radical [CH_3_COOCH_2_CH_3_]^+^; therefore, impurity #2 is ethyl acetate. (3) In Figure 4d, *m*/*z* 100 is the molecular ion peak of CH_3_CH_2_COOCH=CH_2_ and *m*/*z* 57, *m*/*z* 43, and *m*/*z* 29 are the fragment ions CH_3_CH_2_C≡O^+^, CH_3_C≡O^+^, and CH_3_CH_2_^∙^, respectively, formed after the cleavage of CH_3_CH_2_COOCH=CH_2_; therefore, impurity #3 is vinyl propionate.

**Figure 5:**
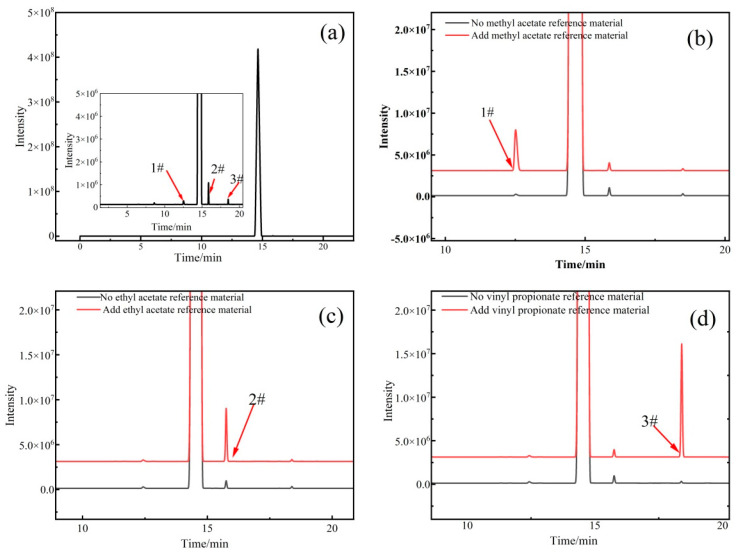
Results of the gas chromatography spike experiment: (**a**) mass spectrogram of vinyl acetate sample; (**b**) comparison before and after adding methyl acetate standard solution to the vinyl acetate CRM; (**c**) comparison before and after adding ethyl acetate standard solution to the vinyl acetate CRM; (**d**) comparison before and after adding vinyl propionate standard solution to the vinyl acetate CRM.

The results of the mass spectrometry analysis were verified by gas chromatography experiments, and the results are shown in Figure 5. Figure 5a shows a typical chromatogram. The retention times of the three impurities were 12.52, 15.86, and 18.49 min. A comparison of these results with the spectra after the addition of methyl acetate, ethyl acetate, and vinyl propionate standard samples indicated that the impurity with a response time of 12.52 min was methyl acetate, the impurity with a response time of 15.86 min was ethyl acetate, and the impurity with a response time of 18.49 min was vinyl propionate (Figure 5b–d), which was consistent with the mass spectrometry results.

#### 2.2.2. Purity Determined by Mass Balance Method

**Figure 6:**
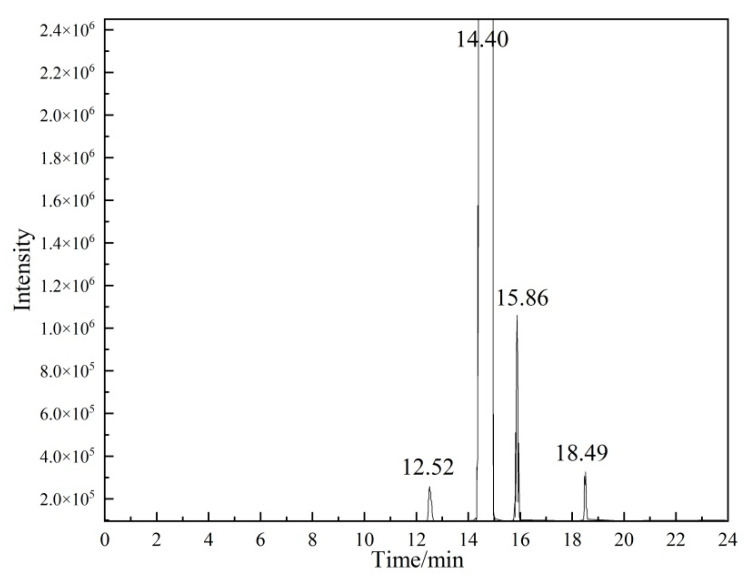
Gas chromatography area normalization of the vinyl acetate CRM.

Figure 6 shows a typical chromatogram of vinyl acetate, in which the main peak at 14.40 min is vinyl acetate and the peaks at 12.52, 15.86, and 18.49 min are impurities. The area normalization method was used to integrate each peak area and to determine the concentration of the analyte. The results are shown in Table 2. The concentration of vinyl acetate was 99.92%. Because of the non-linear response of the GC detector, the results of the area normalization method depended on the magnitude of the response of each component on the detector, and, therefore, a relative correction factor was required in order to correct the area normalization results to achieve more accurate determination.

Calibration solutions were prepared according to the concentration ratio of each component in the vinyl acetate CRM, and were then detected by GC. Relative correction factor fi values of 0.86, 0.83, and 1.23 were calculated for the three impurities according to Equation (3), as shown in Table 2. By substituting the correction factors into Equation (2), the concentration of vinyl acetate was determined to be 99.93%.

(1) $$P\left(\%\right)=\left(1-\sum_{i=1}P_{\mathrm{im}}\right)\left(1-P_{w}-P_{n}-P_{a}\right)\times 100\%$$

(2)
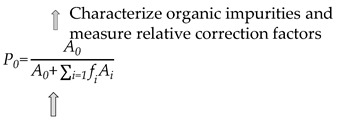

(3) $$f_{i}=\frac{A_{s}\times m_{i}}{A_{i}\times m_{s}}$$

The results regarding the analysis of moisture and organic acids are shown in Table 2. The moisture content was 0.03% with a standard deviation of 0.0015%, the organic acid content was 0.0012% with a standard deviation of 0.00011%, and the content of non-volatile impurities was 0.00016%. As the percentage of non-volatile impurities was very low, it could not affect the initial assignment or uncertainty evaluation of vinyl acetate, so the effect of these impurities was ignored. Finally, the purity of the vinyl acetate CRM, using by the mass balance method (Equation (1)), was determined to be 99.90%.

### 2.3. Homogeneity and Stability Test

The homogeneity results for the vinyl acetate CRM are summarized in Table 3. The data were estimated using analysis of variance (F-test) according to ISO Guide 35:2017 [37], as shown in Table 4. The mean square value between the ${S_{1}}^{2}$ groups and the mean square value within the $S_{2}^{2}$ groups were calculated, and the corresponding F value was 2.01, which was smaller than the critical value F crit (2.04), indicating that the homogeneity of the vinyl acetate CRM did not differ significantly during the period of the experiment. The uncertainty $u_{\mathrm{bb}}$ of uniformity was calculated using Equation (4).

(4) $$u_{\mathrm{bb}}=\sqrt{\frac{\left({S_{1}}^{2}-S_{2}^{2}\right)}{n}}$$

where ${S_{1}}^{2}$ is the mean square error between groups, $S_{2}^{2}$ is the mean square error within a group, and *n* is the number of measurements.

**Figure 7:**
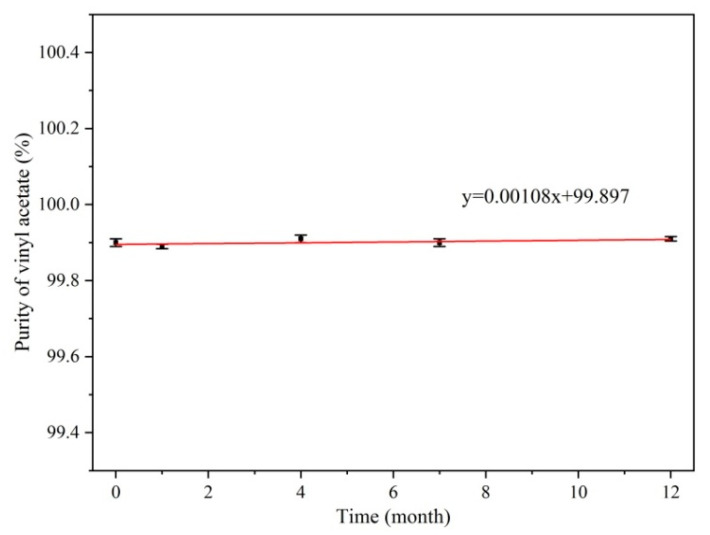
Stability test results.

In the stability test, a vinyl acetate CRM was continuously monitored for 12 months during storage, and the relationship between the measurement results and the monitoring time is shown in Figure 7. The measurement results were linearly fitted to show the stability trend, and regression analysis statistical tests were performed on the data. According to ISO guideline 35:2007 [38], the t test was used to calculate the significance of the slope *a*, where *s*(*a*) is the uncertainty of the slope *a*, as calculated using Equations (5) and (6).

(5) $$s^{2}=\frac{\sum_{1}^{n}{\left(y_{i}-b-ax_{i}\right)}^{2}}{n-2}$$

(6) $$s_{\left(a\right)}=\frac{s}{\sqrt{\sum_{1}^{n}{\left(x_{i}-\overset{-}{x}\right)}^{2}}}$$

$t_{0.95,3}$ is the critical *t*-value at a 95% confidence level and three degrees of freedom. The calculation result satisfied $\left|a\right|<t_{3}^{0.05}\times s_{\left(a\right)},$ and no significant change in the stability was found under long-term storage and simulated transport conditions. The results indicate that the CRM can be stored stably at −4 °C for 12 months. The uncertainty (us) was calculated using Equation (7), where T represents the duration of the long-term stability study.

(7) $$u_{s}=s_{\left(a\right)}\times T$$

### 2.4. Uncertainty Estimation

#### 2.4.1. Uncertainty of the Mass Balance Method

According to the ISO Guidelines for the Expression of Uncertainty in Measurement [39], the uncertainty of the mass balance method $u_{\mathrm{MB}}$ mainly comes from principal component measurement $u\left(P_{0}\right)$, moisture measurement $u\left(x_{w}\right)$, and organic acid measurement. The detection $u\left(x_{a}\right)$ and the measurement $u\left(x_{n}\right)$ of less volatile impurities can be evaluated using Equation (8).

(8) $$u_{\mathrm{MB}}=P_{\mathrm{MB}}\;\times \sqrt{{u\left(P_{0}\right)}^{2}+\frac{u{\left(x_{w}\right)}^{2}+u{\left(x_{a}\right)}^{2}+u{\left(x_{n}\right)}^{2}}{{\left(1-x_{W}-x_{a}-x_{n}\right)}^{2}}}$$

For the main components analyzed by GC-FID, the combined uncertainty *u*(*P*_0_) can be evaluated as:

(9) $${u\left(P_{0}\right)}_{\;}=\sqrt{{u_{\mathrm{rel},1}}^{2}+{u_{\mathrm{rel},2}}^{2}}\;$$

where $u_{\mathrm{rel},1}$ represents the uncertainty introduced by the repeatability of the measurement, which is usually equal to the relative standard deviation of the measurement, and $u_{\mathrm{rel},2}$ represents the uncertainty introduced by the limit of detection (LOD) of GC-FID.

The uncertainty in the water measurement $u\left(x_{w}\right),$ using the Karl Fischer coulomb method, can be calculated by the following formula:

(10) $$u\left(x_{w}\right)=x_{w}\sqrt{u_{1}^{2}+{\left[\frac{u\left(m\right)}{m}\right]}^{2}+{\left[\frac{u\left(W\right)}{W}\right]}^{2}+{\left[\frac{u\left(f\right)}{f}\right]}^{2}}$$

where $u_{1}$ represents the relative standard uncertainty introduced by measurement repeatability; $u\left(m\right)$ represents the uncertainty of the sample’s quality; $u\left(W\right)$ represents the standard uncertainty of the quality of water; and *u*(*f*) represents the uncertainty of the correction factor, calculated from the uncertainty of the moisture reference material.

The uncertainty $u\left(x_{a}\right)$ introduced by the titration method for the organic acid measurement was equal to the relative standard deviation of the repeated determinations.

The calculation results for the various uncertainties are shown in Table 5, and the uncertainty introduced by the mass balance method was 0.105%.

#### 2.4.2. Combined Uncertainty

According to ISO guideline 35, the combined uncertainty $u_{\mathrm{CRM}}$ for a vinyl acetate CRM includes the uncertainty in the homogeneity test; the uncertainty in the stability study; and the uncertainty in the fixed value, as calculated from Equation (11) [32].

(11) $$u_{\mathrm{CRM}}=\sqrt{u_{\mathrm{bb}}^{2}+u_{\mathrm{ls}}^{2}+u_{\mathrm{MB}}^{2}}$$

where $u_{\mathrm{bb}}\;$is the uncertainty of the uniformity test, $u_{\mathrm{ls}}$ is the uncertainty of the stability test, and $u_{\mathrm{MB}}$ is the uncertainty of the mass balance legal value. Table 6 summarizes the sources of uncertainty and the results of the assessment. When the confidence probability was 95%, the expansion factor was 2, and the expansion uncertainty $U_{\mathrm{CRM}}$ of the vinyl acetate CRM was 0.3%.

## 3. Materials and Methods

### 3.1. Apparatus and Materials

Low-resolution mass spectrometry (Agilent 6890N-5973N GC/MS, USA), high-resolution mass spectrometry (Agilent 7890A/7200 Q-TOF GC/MS, USA), superconducting nuclear magnetic resonance spectroscopy (Bruker AV-500, USA), and Fourier transform infrared spectrometry (Thermo Fisher, Waltham, MA, USA, Thermo Nicolet In10MX-Iz10, USA) were used to characterize the CRM candidate; gas chromatography was also performed on an Agilent 6890N-5973N GC/MS instrument (Agilent Technologies, Santa Clara, CA, USA) equipped with a flame ionization detector (FID). The present study also employed a Karl Fischer automatic moisture analyzer (Mettler C30, Mettler Toledo, Zurich, Switzerland), an inductively coupled plasma mass spectrometer (Agilent 7500a, USA), and a potentiometric titrator (Mettler T50, Mettler Toledo, Switzerland).

The CRM candidate material for vinyl acetate required for the experiment was commissioned from the Beijing Oriental Organic Chemical Factory. Methyl acetate, ethyl acetate, and vinyl propionate were all standard products produced by Dr. Ehrenstorfer, Germany. The Karl Fischer reagent (without pyridine) had a titer of approximately 2–5 mg/mL. All other reagents used were of analytical grade or higher.

### 3.2. Methods

#### 3.2.1. Preparation of CRM Candidate

Because of the instability and toxicity of vinyl acetate, the raw materials for vinyl acetate used in this study were prepared by the Beijing Dongfang Organic Chemical Factory (Beijing, China), the main information about the reagents can be obtained from the Table S1.

Preparation method: Acetic acid, ethylene, and oxygen underwent a gas phase reaction under the action of a catalyst. After the reaction was completed, the obtained mixture was purified by rectification three times, and finally, vinyl acetate raw material was obtained. The material was cooled to −18 °C and dispensed into 2 mL brown ampoules under nitrogen protection, and the ampoules were immediately sealed. The same batch of vinyl acetate feedstock was used to fill 450 vials of CRM candidate before being stored at 4 °C.

#### 3.2.2. Characterization of the CRM Candidate

(1) MS analysis

The CRM candidate was characterized by MS, and the molecular weights were determined. A 250 μL sample of CRM was introduced to a DB-5MS capillary column (30 m × 0.25 mm × 0.25 μm) for separation. The experimental conditions were as follows: The carrier gas was helium; the flow rate in constant flow mode was 1 mL/min; the inlet temperature was 200 °C; the ion source temperature was 230 °C; the quadrupole temperature was 150 °C; the transfer line temperature was 250 °C; the split ratio was 100:1; the ionization method was electron bombardment ionization (EI) (70 eV); and the heating program progressed from an initial temperature of 35 °C to the target temperature over 10 min. The range of mass *m*/*z* = 30–350 was scanned using a one-level full scan.

(2) FT-IR

FT-IR spectroscopy was used to characterize the structures of the CRM candidate. Samples were prepared using the liquid film method, and infrared spectra were collected using the attenuated total reflection (ATR) technique using the infrared wavelength of polystyrene as a reference. All infrared spectra were acquired in the range of 4000–400 cm^−1^ at a resolution of 4.000 cm^−1^.

(3) NMR

A sample of the CRM candidate (0.5 mg) was weighed and dissolved in 0.50 mL of CDCl_3_, and the proton and carbon spectra were acquired by means of superconducting NMR.

#### 3.2.3. Mass Balance Method

The mass balance method involves the measurement of the major components in a sample, as well as moisture, less volatile impurities, and organic acids. According to the content of each component, the purity of vinyl acetate can be calculated by Equation (12) [40].
(12)
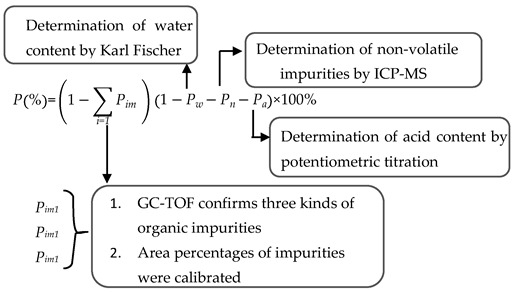

where *P_im_*, *P_w_*, *P_n_*, and *P_a_* are the contents of organic impurities, water, non-volatile impurities, and organic acids, respectively, in the raw material.

(1) Qualitative analysis of organic components

The organic impurities in vinyl acetate were characterized by high-resolution mass spectrometry, and the results were verified by gas chromatography. The experimental conditions for the high-resolution mass spectrometry were the same as those described in Section 3.2.2 (1).

For the GC conditions (the instrument model used was an Agilent 6890N-5973N GC/MS), 1 μL of vinyl acetate solution was separated using a J&W DB-624 capillary column (60 m × 0.32 mm × 0.25 μm). The experimental conditions were as follows: carrier gas helium; flow rate of 1 mL/min in constant flow mode; inlet temperature of 200 °C; ion source temperature of 230 °C; quadrupole temperature of 150 °C; transfer line temperature of 200 °C; and split ratio of 10:1. Temperature programming conditions were as follows: The initial temperature was 40 °C, the temperature was raised to 50 °C at a rate of 2 °C/min, and then the temperature was raised to 140 °C at a rate of 5 °C/min before being maintained for 1 min.

(2) Determination of organic components

The gas chromatography area normalization method was used to measure the contents of organic components in the CRM candidate, and the experimental conditions were the same as those described in Section 3.2.3 (1). The organic component after introducing the correction factor can be calculated from Equation (13).

(13) $$P_{0}=\frac{A_{0}}{A_{0}+\sum_{i=1}f_{i}A_{i}}$$

where *P*_0_ is the content of the main component; *A*_0_ represents the peak areas of the main components; and *f_i_*, *A_i_* (*i* = 1 − n) represent the relative correction factors and peak areas of the organic impurities, respectively. The experimental conditions were the same as those used for the gas chromatography spiked experiment.

(14) $$f_{i}=\frac{A_{s}\times m_{i}}{A_{i}\times m_{s}}$$

where *A_s_* is the peak area of the standard substance, *A_i_* is the peak area of impurity *i*, *m_s_* is the mass of the standard substance, and *m_i_* is the mass of impurity *i*.

(3) Determination of water

Because vinyl acetate is prone to polymerization when heated, the Karl Fischer coulometric method was chosen to measure the water content in the CRM candidate. First, 100 mL of Karl Fischer reagent was added into the calibrated instrument, the vibration frequency of the oscillator was set to 155 r/min, and the solution was mixed evenly. Then, approximately 1 mL of vinyl acetate CRM was added and reacted to the end point under the protection of nitrogen. A blank experiment was performed to ensure the accuracy of the test data.

(4) Determination of inorganic impurities

The mass fraction of inorganic impurities in the CRM candidate was measured using ICP-MS. The instrument parameters were as follows: the radio frequency power was 1300 W; the carrier gas flow rate was 1.20 L/min; the sampling rate was 0.1 r/s; and the measurement was repeated three times in the full quantitative analysis mode. The measurement method was as follows: 1 mL of candidate CRM was transferred to a 10 mL volumetric flask, diluted to volume with deionized water, shaken well, and used directly for ICP-MS.

(5) Determination of acid

A Mettler T50 potentiometric titrator was used to measure the content of organic acids. The measurement method was as follows: 1.5 mL of vinyl acetate solution was added to the potentiometric titrator, and calibrated sodium hydroxide solution was automatically added dropwise to the titration end point. To ensure the accuracy of the experimental data, a DG113-SC non-aqueous titration composite electrode was used to monitor the titration end point.

### 3.3. Homogeneity and Stability Test

According to the packing sequence, a total of 15 bottles of samples were randomly selected, with five bottles of vinyl acetate CRM candidate samples taken from each of the front, middle, and back. Each bottle was tested three times using the established GC-FID method. One-way analysis of variance (ANOVA) was used to test the homogeneity of the sample values. Comparison of the calculated value of F with the critical value of F (with a confidence level of 95%) determined whether there was a significant difference in homogeneity.

Because of the unstable nature of vinyl acetate, self-polymerization reactions may occur during long-term storage to affect the purity; therefore, monitoring the stability of vinyl acetate CRMs is the key to ensuring the quality of the measurements. The packaged vinyl acetate CRM candidate products were stored at 4 °C in the dark. Following the principle of dense first and thin later, and the concentrations were measured after 0, 1, 4, 7, and 12 months of storage. The purity of the vinyl acetate CRM products was measured by GC-FID, and the samples were measured three times at each time point.

#### Statistical Analyses

The homogeneity test data were analyzed by ANOVA (F-test). The F-value was calculated according to Equation (15), where $S_{1}^{2}$ is the mean square error between groups and $S_{2}^{2}$ is the mean square error within a group.

(15) $$F=\frac{S_{1}^{2}}{S_{2}^{2}}$$

The stability studies were assessed by performing ANOVA (*t*-test) on linear regression data to determine the stability trends in CRM candidate purity. Statistical tests for regression analysis were performed on the stability measurements. We compared the absolute value of the slope (a) with the product of t the critical value and the slope uncertainty s(a). The calculation of s(a) is shown in Equations (5) and (6), which are mentioned in Section 2.3.

## 4. Conclusions

In this experiment, the raw materials were processed and produced, following the existing production process, and the filling and heat-sealing procedures were strictly controlled to prepare the vinyl acetate CRM. GC-MS, FT-IR, and NMR spectrometry were used to characterize the CRM candidate. Gas chromatography tandem high-resolution mass spectrometry indicated that the organic impurities in the vinyl acetate candidate were methyl acetate, ethyl acetate, and vinyl propionate. The correction factors for the three impurities were calculated, and the concentrations of the main components were determined by the gas chromatographic area normalization method. The mass balance method was used to determine the purity of vinyl acetate, and the extended uncertainty was calculated. A homogeneity and stability test indicated that the vinyl acetate CRM had good homogeneity and could be stored stably for 12 months. This study will rectify the lack of available vinyl acetate purity reference materials in China, provides a method for the comprehensive purity analysis of vinyl acetate, and can be used to develop certified reference materials with metrological traceability to SI units, and vinyl acetate certified reference material classification certificate available in File S2. 

## Figures and Tables

**Table 1:** High-resolution mass spectrometry data on three impurities in vinyl acetate.

Impurity No.	Retention Time	Measured Value (*m*/*z*)	Theoretical Value (*m*/*z*)	Elemental Composition
Vinyl acetate	2.25 min	86.0366	86.0362	C_4_H_6_O_2_
		43.0177	43.0178	C_2_H_3_O
Impurity #1	1.96 min	74.0361	74.0362	C_3_H_6_O_2_
		59.0124	59.0128	C_2_H_3_O_2_
		43.0177	43.0178	C_2_H_3_O
Impurity #2	2.59 min	88.0520	88.0519	C_4_H_8_O_2_
		70.0410	70.0143	C_4_H_6_O
		61.0281	61.0284	C_2_H_5_O_2_
		43.0174	43.0178	C_2_H_3_O
Impurity #3	3.71 min	100.02	100.02	C_5_H_8_O_2_
		57.0337	57.0335	C_3_H_5_O
		43.0176	43.0178	C_2_H_3_O
		29.0382	29.0386	C_2_H_5_

**Table 2:** Purity of the analytes, determined using the mass balance method.

Measurement	Retention Time (min)	*A_i_*	Concentration (%)	*f_i_* (%)	Concentration (Calibration %)	RSD (%)
Methyl acetate	12.52	9,315,686	0.02	0.86	--	--
Ethyl acetate	15.86	33,948,215	0.05	0.83	--	--
Vinyl propionate	18.49	7,934,083	0.01	1.23	--	--
Vinyl acetate (*P*_0_)	14.40	63,973,339,424	99.92	--	99.93	0.0029
$x_{w}$	0.030					0.0015
$x_{a}$	0.0012					0.00011
*P_MB_*	99.0					0.018

**Table 3:** Homogeneity results for the vinyl acetate CRM (%).

Number	1	2	3	Means
1	99.89	99.88	99.90	99.89
2	99.89	99.87	99.91	99.89
3	99.87	99.90	99.89	99.89
4	99.87	99.93	99.90	99.90
5	99.90	99.91	99.93	99.91
6	99.91	99.89	99.88	99.89
7	99.91	99.90	99.92	99.91
8	99.90	99.87	99.88	99.88
9	99.93	99.91	99.93	99.92
10	99.91	99.88	99.90	99.90
11	99.90	99.88	99.89	99.89
12	99.89	99.90	99.90	99.90
13	99.88	99.89	99.89	99.89
14	99.92	99.90	99.92	99.91
15	99.90	99.92	99.90	99.91
Overall mean	99.90			
Standard deviation	0.017			

**Table 4:** ANOVA analysis of homogeneity results.

Parameters	Values
Mean square between groups	$S_{1}^{2}$ = 0.00043
Mean square within groups	$S_{2}^{2}$ = 0.00022
F	F = $S_{1}^{2}$/$S_{2}^{2}$ = 2.01
F_0.05_(14, 30)	2.04
Conclusion	$F<F_{0.05}(14,\;30)$

**Table 5:** Uncertainties of the parameters in the mass balance method.

Uncertainty Symbols	$u\left(x_{w}\right)$	$u\left(x_{a}\right)$	$u_{rel,1}$	$u_{rel,2}$	$u\left(P_{0}\right)$	$u_{\mathrm{MB}}$
Results	0.0015%	0.00011%	0.009%	0.105%	0.105%	0.105%

**Table 6:** Uncertainty evaluation of vinyl acetate CRM.

Uncertainty Symbols	Uncertainty Sources	Results
$u_{\mathrm{bb}}$	Homogeneity test	0.00849%
$u_{\mathrm{ls}}$	Long-term stability study	0.00930%
$u_{\mathrm{MB}}$	Mass balance method	0.105%
$u\left(P_{0}\right)$	GC-FID	0.105%
$u_{\mathrm{CRM}}$	Combined uncertainty	0.11%
$U_{\mathrm{CRM}}$	Expanded combined uncertainty	0.3%

## Data Availability

Data sharing is not applicable.

## Supplementary Materials

The following supporting information can be downloaded at: https://www.mdpi.com/article/10.3390/molecules28176245/s1, Table S1: Concentration of some domestic and foreign vinyl acetate reagent labels; File S2: Vinyl Acetate certified reference material classification certificate.

## Sample Availability

Samples of the compounds are not available from the authors.
