# Supplementary material for: Development of a Purity Certified Reference Material for Vinyl Acetate
Source: Molecules. 2023 Aug 25;28(17):6245. doi: 10.3390/molecules28176245 (PMC10488496; doi:10.3390/molecules28176245)
Supplement: Supplementary file 1 [file molecules-28-06245-s001.zip › molecules-2553424-supplementary/English version of Vinyl Acetate certified reference material classification certificate.pdf]

The Gradation Certificate of the National Certified Reference Material

This is to certify that the following reference materials have been approved, according to "the Law on Metrology of the People's Republic of China", as the Second class of National Certified Reference Materials in compliance with the requirements of the "Regulation of Reference Materials".

Name of the Reference Material: Vinyl Acetate Certified Reference Material

Code(s): GBW(E)062710

Producers of the Reference Material: China Tobacco Zhengzhou Tobacco Academe

Date issued: April 26, 2020

Table of Certified Value(s)

Name	Code(s)	Quality Score ($\times 10^{-2}$)	Relative Uncertainty (%)
Vinyl Acetate Certified Reference Material	GBW(E)062710	99.9	0.3
