# Supplementary material for: Development of a Purity Certified Reference Material for Vinyl Acetate
Source: Molecules. 2023 Aug 25;28(17):6245. doi: 10.3390/molecules28176245 (PMC10488496; doi:10.3390/molecules28176245)
Supplement: Supplementary file 1 [file molecules-28-06245-s001.zip › molecules-2553424-supplementary/File S1 - Vinyl Acetate Reagents from Different Manufacturers.pdf]

During the process of development for vinyl acetate certified reference material (CRM), we have investigated the vinyl acetate reagents prepared by several producers with the purity values shown in Table S1.

Table S1: Concentration of some domestic and foreign vinyl acetate reagent labels

	Reagent No	Manufacturer	Information for the reagents of vinyl acetate		
			Marking purity	Method	Water (%)
Vinyl acetate CAS:108-05-4	1	China J&K Company	>99%	GC-FID	0.0013
	2	Thermo fisher Acros Company	>99%	GC-FID	0.0014
	3	Alfa Aesar Company	>99%	GC-FID	0.0040
	4	TCI Company	>99%	GC-FID	0.0045
	5	China Sinopharm Company	>99.5%	GC-FID	0.0040
	6	Beijing Dongfang Organic Chemical Factory	>99.5%	GC-FID	0.0014

The results of the vinyl acetate certified reference material developed by the authors are shown in Table S2.

Table S2: Vinyl acetate CRM purity and impurity content

Measurement	Concentration (calibration %)
Methyl acetate	0.02
Ethyl acetate	0.05
Vinyl propionate	0.01
Water	0.030
Acetic acid	0.0012
Vinyl acetate (P_{MB})	99.90
Expanded combined uncertainty	0.3%
