# Supplementary material for: Development of a Purity Certified Reference Material for Vinyl Acetate
Source: Molecules. 2023 Aug 25;28(17):6245. doi: 10.3390/molecules28176245 (PMC10488496; doi:10.3390/molecules28176245)
Supplement: Supplementary file 1 [file molecules-28-06245-s001.zip › molecules-2553424-supplementary/Vinyl Acetate certified reference material classification certificate.pdf]

国家标准物质定级证书

The Gradation Certificate of the National Certified Reference Material

[2020] 国标物 证字第 2173 号

根据《中华人民共和国计量法》，按照《标准物质管理办法》的要求，经鉴定，批准为国家 二 级标准物质，特发此证。

This is to certify that the following reference materials have been approved, according to "the Law on Metrology of the People's Republic of China", as the Second class of National Certified Reference Materials in compliance with the requirements of the "Regulation of Reference Materials".

标准物质名称 ：乙酸乙烯酯纯度标准物质
Name of the Reference Material

编号 ：GBW（E）062710
Code(s)

标准物质研制机构 ：中国烟草总公司郑州烟草研究院
Producers of the Reference Material

国家市场监督管理总局

发证机关（印章）
The Issuing Authority

发证日期 2020年 4月 26日
Date Issued April 26, 2020

定值数据表/Table of Certified Value(s)

名称	编 号	质量分数 ($\times 10^{-2}$)	相对不确定度 (%)
乙酸乙烯酯纯度标准物质	GBW（E）062710	99.9	0.3

研制单位：中国烟草总公司郑州烟草研究院（郑州市）
